# Systematic assessment of clinical and bacteriological markers for tuberculosis reveals discordance and inaccuracy of symptom-based diagnosis for treatment response monitoring

**DOI:** 10.3389/fmed.2022.992451

**Published:** 2022-10-28

**Authors:** Bariki Mtafya, Issa Sabi, Joseph John, Emanuel Sichone, Wilyhelmina Olomi, Stephen H. Gillespie, Nyanda E. Ntinginya, Wilber Sabiiti

**Affiliations:** ^1^National Institute for Medical Research, Mbeya Medical Research Centre, Mbeya, Tanzania; ^2^School of Medicine, University of St Andrews, St Andrews, United Kingdom

**Keywords:** TB-MBLA, diagnosis, monitoring, TB symptoms, bacteriological tests

## Abstract

**Background:**

Clinical symptoms are the benchmark of tuberculosis (TB) diagnosis and monitoring of treatment response but are not clear how they relate to TB bacteriology, particularly the novel tuberculosis-molecular bacterial load assay (TB-MBLA).

**Methods:**

Presumptive cases were bacteriologically confirmed for TB and assessed for symptoms and bacteriological resolution using smear microscopy (SM), culture, and TB-MBLA over 6-month treatment course. Kaplan–Meier and Kappa statistics were used to test the relationship between symptoms and bacteriological positivity.

**Results:**

A cohort of 46 bacteriologically confirmed TB cases were analyzed for treatment response over a 6-month treatment course. Pre-treatment symptoms and bacteriological positivity concurred in over 70% of the cases. This agreement was lost in over 50% of cases whose chest pain, night sweat, and loss of appetite had resolved by week 2 of treatment. Cough resolved at a 3.2% rate weekly and was 0.3% slower than the combined bacteriological (average of MGIT and TB-MBLA positivity) resolution rate, 3.5% per week. A decrease in TB-MBLA positivity reflected a fall in bacillary load, 5.7 ± 1.3- at baseline to 0.30 ± 1.0- log_10_ eCFU/ml at month 6, and closer to cough resolution than other bacteriological measures, accounting for the only one bacteriologically positive case out of seven still coughing at month 6. Low baseline bacillary load patients were more likely to be bacteriologically negative, HR 5.6, *p* = 0.003 and HR 3.2, *p* = 0.014 by months 2 and 6 of treatment, respectively.

**Conclusion:**

The probability of clinical symptoms reflecting bacteriological positivity weakens as the patient progresses on anti-TB therapy, making the symptom-based diagnosis a less reliable marker of treatment response.

## Introduction

Tuberculosis (TB) is the leading cause of death attributed to a single microbial pathogen worldwide, ranking above HIV/AIDS until the recent coronavirus pandemic (1). In 2020, around 10 million people developed TB disease and 1.4 million died (2). The current 6-months TB treatment with Isoniazid (H), Rifampicin (R), Pyrazinamide (P), and Ethambutol (E) regimens is effective in healthcare settings but can be hampered by low adherence and long treatment duration that can be challenging to finish (3–5). Accurate and rapid tests are required to help clinicians to identify patients at risk of treatment failure including those who may require an extended treatment or treatment change (6). The availability of such tools would reduce treatment costs and expedite the evaluation of new TB medicines (7).

Currently, diagnosis and treatment monitoring relies on the initial assessment of TB symptoms followed by bacteriological confirmation in clinical specimens (8, 9). Presence of TB symptoms such as cough, fever, night sweats, hemoptysis, sputum production, and weight loss are associated with active TB disease (9, 10). Despite the technical difficulties to screen TB symptoms and its low sensitivity and specificity (11), a combination of three symptoms (cough, fever, and sweats) demonstrated a 93% sensitivity and 36% specificity for active TB in people living with Human Immune Deficiency Virus (PLHIV) (12). Previous clinical trials showed the association of cough, fever, and night sweats with TB bacillary burden at diagnosis and relapse (13, 14). Thus, careful assessment of TB symptoms may be an alternative diagnostic and monitoring tool in healthcare settings with limited capacity to perform bacteriological tests.

Bacteriological tests are the reference standard for the diagnosis of active TB and monitoring. Tests detecting DNA of *Mycobacterium tuberculosis* (*M.tb*) such as the Xpert MTB/RIF Assay are rapid and sensitive for diagnosis but are not useful for monitoring (15) as DNA can be detected from both live and dead cells (16, 17). Reliance on standard smear microscopy (SM) and culture methods have many limitations for monitoring (18–22). Sputum smear is cheap and easy to perform but has low sensitivity and specificity (23, 24). Therefore, in the absence of the WHO-recommended tools, many clinicians use clinical symptoms as the means for monitoring treatment response (24, 25).

Tuberculosis-molecular bacterial load assay (TB-MBLA) is a promising test for treatment response monitoring. It quantifies 16S rRNA of *M.tb* by reverse transcriptase polymerase chain reaction as the marker viable *M.tb* (26–28). Early studies have consistently retrieved *M.tb* 16S rRNA in patient samples reflecting viable bacilli during treatment with a strong correction with standard MGIT culture (29–32). In this study, we prospectively assessed clinical measures of patient improvement compared to standard-of-care bacteriological tests and with the novel TB-MBLA before and throughout 6 months of standard TB therapy.

## Materials and methods

### Ethics

The study was approved by the Mbeya Medical Research and Ethics Committee (MRH/R.10/18VOLL.VII/12) and the University of St Andrews Teaching and Research Ethics Committee (MD12678). National approval was obtained from the National Institute for Medical Research (NIMR/HQ/R.8a/Vol.IX/2400) in Tanzania. All participants provided written consent or witnessed verbal consent for those who could not write or read.

### Study design

This was a longitudinal prospective study assessing the relationship of TB symptoms with bacteriological measures before and during standard 6 months of TB therapy in routine healthcare settings.

### Study sites

The study was conducted in Mbeya, Tanzania from January 2017 to March 2018. Patients were enrolled from the Mbeya Zonal Referral Hospital (MZRH), Mbeya Regional Referral Hospital (MRRH), and Mbeya Rural District Hospital (MRDH). Mbeya is a high TB burden border region and major transport gateway to Malawi, Zambia, and the Democratic Republic of Congo.

### Patient recruitment and selection criteria

Patients with presumed symptoms of pulmonary TB (PTB) and those who had at least one of the following symptoms; cough, night sweats, chest pain, loss of appetite, and weight loss aged between 18 and 65 years were selected to provide sputum specimens for initial diagnosis with Xpert MTB/RIF Assay or sputum SM at the health facility. Xpert MTB/RIF Assay positive who tested negative to RIF resistance were given permission to participate in the study, which included providing follow-up samples for the bacteriological assessment of treatment response. Patients who were severely ill, unable to produce sputum, and those who were more than 50 km from a health facility, making it difficult for them to attend all follow-up visits, were excluded from the study.

### Bacteriological confirmation

**FIGURE 1:**
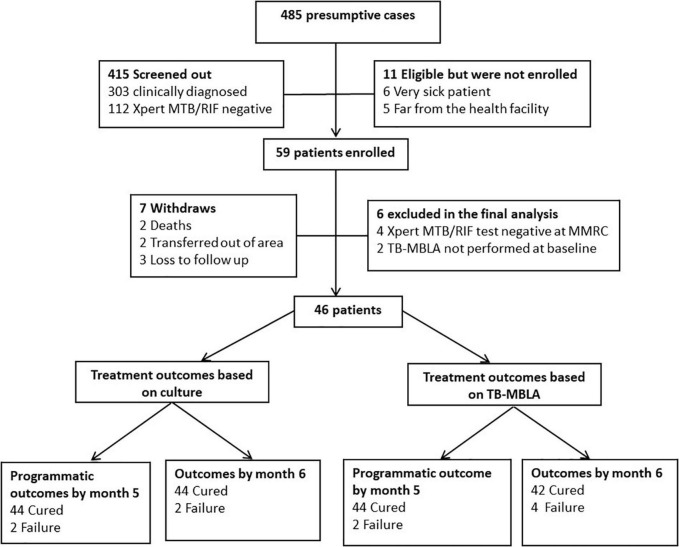
Screening workflow, enrollment, follow-up, and final outcomes. Three hundred and three patients were screened out because they were bacteriological test negative at diagnosis and were treated clinically. Of the 59 enrolled patients, seven patients withdrew from the study (two died, three transferred out of the area, and three were lost to follow-up). Six patients were excluded from the final analysis (four due to inconsistent Xpert MTB/RIF results at the health facility and confirmatory Xpert MTB/RIF test at the NIMR-MMRC testing laboratory and two patients had low sputum volume for TB-MBLA at baseline). Three patients were lost to follow-up and two patients who were HIV positive died.

Prior to the initiation of standard TB therapy based on diagnostic results at the health facility, all consented patients provided a second pre-treatment sputum sample for bacteriological confirmation at NIMR-Mbeya Medical Research Centre (NIMR-MMRC) using sputum SM and Xpert MTB/RIF Assay, Culture and novel TB-MBLA. Patients who were bacteriologically negative or with the discordant confirmatory result at NIMR-MMRC including those who were treated based on the clinician’s judgment were excluded in the study (Figure 1). All patients received standard TB therapy consisting of H-Z-R-E for 2 months followed by 4 months with H-R.

### Treatment response monitoring and study outcomes

Participants were monitored for symptom and bacteriological positivity at weeks 2, 8, 22, and 26 of standard TB therapy. TB symptoms such as cough, chest pain, sputum production, loss of appetite, and night sweats were assessed at diagnosis, and all treatment follow-up visits by a clinician or a TB and leprosy coordinator at the healthcare facility prior to the collection of sputum specimens for bacteriological tests. Bacteriological response during treatment was measured using standard ZN microscopy, culture, and the novel TB-MBLA.

The study outcomes were the resolution of TB clinical symptoms and bacteriological conversion from positive to negative to inform the treatment continuation phase at week 8 and to establish final outcomes at the end of treatment weeks 22 and 26. Assessment of the final study outcomes was performed using standard SM, culture and results compared to the novel TB-MBLA. TB cured patients were defined as PTB patients with bacteriologically confirmed at diagnosis who converted to smear or culture negative in the last month of treatment and on at least one previous occasion (33).

### Bacteriological tests

Sputum samples were homogenized using a sterile magnetic stirrer for 30 min and 1 ml of aliquot was mixed with 4 ml of guanidine thiocyanate (GTC) and used for TB-MBLA. Total RNA extraction for TB-MBLA was performed using the FastRNA Pro Kit (MP Biomedicals) and removal of genomic DNA was done using Turbo DNA free kit (Ambion). Reverse transcriptase-quantitative polymerase chain reactions (RT-qPCR) were performed on the Rotor-Gene Q machine (Qiagen) using the QuantiTect PCR NoROX mix (Qiagen) and TaqMan dual labeled probes (Eurofins Genomics). Using a pre-developed standard curve, the RT-PCR quantification cycle (Cq) values were converted to bacterial load (BL) and reported as estimated colony-forming units per/ml of sputum (eCFU/ml) as previously described (27, 34). The standard curve was developed using RNA extracted from mycobacterial cells with known colony-forming units per ml (CFU/ml).

A total of more than 2 ml of sputum was decontaminated with 1% of *N*-acetyl-L-cysteine combined with 2% of sodium hydroxide for 20 min. Concentrated sputum pellets were used for ZN microscopy, MGIT liquid culture (mycobacterial growth indicator tubes), and solid culture (LJ) (Beckton and Dickson Company, MD, USA) following the manufacturer’s instructions. The growth of *M.tb* in culture was confirmed by ZN stain, lateral flow antigen test (MPT64, Beckton and Dickson Company, MD, USA), and blood agar plate (BAP) to confirm the purity of culture (35). Antimicrobial drug susceptibility tests for standard regimens (H-Z-R-E) were performed in the BACTEC MGIT 960 System following manufacturer instructions (36).

Treatment outcome was assessed at weeks 22 and 26 of treatment using the culture as the gold standard compared to TB-MBLA positivity at this stage of treatment. To assess the effect of baseline BL on the resolution of TB symptoms during treatment, we computed the median BL at baseline and stratified the patients into “High BL” for those with BL greater than the median value and “Low BL” for those with BL below the median value. Additionally, we stratified patients based on HIV status to determine if HIV has an impact on the resolution of TB symptoms. The average percent positivity of the most sensitive bacteriological tests, MGIT culture, and TB-MBLA were used to calculate the bacteriological positivity score.

### Statistical analyses

Data were analyzed using STATA software (version 14) and GraphPad Prism version 9.3.1 (GraphPad Software, La Jolla, CA, USA). Multivariate hazard ratio was used to estimate the association between baseline BL and time to the resolution of TB symptoms from the start of TB therapy. Cox proportion hazard ratio was used to determine the association between the median time to the resolution of TB symptoms stratified by baseline BL or HIV status. Fischer exact test was used to assess the association of TB symptoms with bacteriological tests at week 8, exploring whether TB symptoms may be used to inform treatment continuation phase as for standard culture and SM. Kaplan–Meier curves were used to calculate the probability of TB bacillary load clearance at weeks 8 and 26 of treatment and Kappa statistics to assess percentage agreement between TB symptoms and bacteriological tests. All statistical analyses were considered significant at a *p*-value of less than 0.05.

## Results

### Patients’ characteristics

Figure 1 depicts the flow of screened patients, enrollment, and follow-up. Of the 485 presumptive patients screened, 59 (12.8%) were enrolled in the study and 13 (22.03%) were excluded from the analysis because they either did not complete follow-up or their initial Xpert MTB/RIF Assay result at the health facility was discordant with the second confirmatory MTB/RIF Assay test performed NIMR-MMRC. As a result, 46 patients with a total of 230 serial samples collected longitudinally during the 6 months of standard treatment period were analyzed. Median age (range) was 37 years (18–65), 60.9% (28/46) were male subjects, 39.1% (18/46) were HIV positive, and 13% (6/46) were re-treatment cases (Table 1). Using the WHO definition of cure, 4.3% (2/46) each at months 5 and 6 were MGIT culture positive and considered as treatment failure. TB-MBLA concurred with MGIT at month 5 detecting 4.3% (2/46) patients but two more at month 6, 8.6% (4/46). All 46 (100%) patients were phenotypically susceptible to the first-line H-Z-R-E regimen determined by the BD BACTEC MGIT 960 Culture Systems.

**TABLE 1:** Baseline characteristics of the patients (*N* = 46).

Age in years, median (range)	37 (18–65)
**Sex**	
Male, *n* (%)	28 (60.9)
Re-treatment, *n* (%)	6 (13.0)
**HIV status**	
HIV positive, *n* (%)	18 (39.1)
HIV negative, *n* (%)	25 (54.3)
Unknown HIV status, *n* (%)	3 (6.5)
**Bacteriological tests**	
Xpert MTB/RIF Assay, *n* (%)	46 (100)
Sputum smear microscopy, *n* (%)	39 (84.8)
Liquid culture in MGIT, *n* (%)	43 (93.5)
Solid culture in LJ, *n* (%)	39 (84.7)
TB-MBLA, *n* (%)	46 (100)
**TB clinical symptoms**	
Cough, *n* (%)	45 (97.8)
Chest pain, *n* (%)	37 (80.3)
Sputum production, *n* (%)	46 (100)
Sweats, *n* (%)	40 (86.9)
Loss of appetite, *n* (%)	35 (76.1)

HIV, human immunodeficiency virus; LJ, Lowenstein–Jensen media; MGIT, mycobacterium growth indicator tubes; TB, tuberculosis; TB-MBLA; tuberculosis molecular bacterial load assay.

### Resolution of tuberculosis symptoms compared to bacteriological tests positivity throughout treatment

**FIGURE 2:**
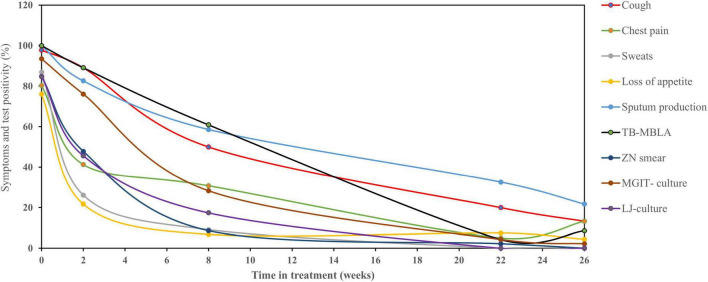
Relationship of TB clinical symptoms clearance with bacteriological tests. Clinical symptoms resolved rapidly during TB therapy with the exception of cough and sputum production which resolved slowly than culture, microscopy, and TB-MBLA. Data are presented as the percentage (%) of patients with clinical symptoms and bacteriological positivity over the time in treatments (weeks).

**FIGURE 3:**
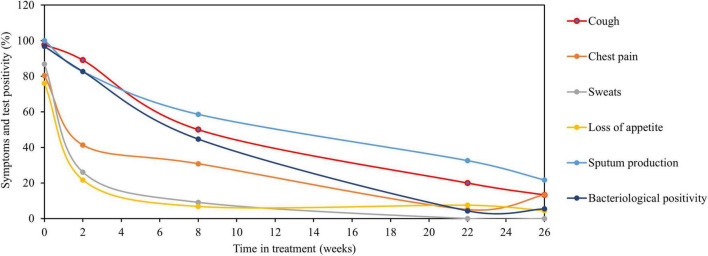
Average bacteriological positivity compared to clinical symptoms clearance. Relationship of clinical symptoms clearance compared to average bacteriological positivity of TB-MBLA and culture. Clinical symptoms (sweats, loss of appetite, and chest pain) resolved more rapidly than the average bacteriological positivity. Resolution of cough was slow matching the resolution of average bacteriological positivity, while sputum production resolved more slowly than average bacteriological positivity. Data are presented as the percentage (%) of patients with clinical symptoms and average bacteriological positivity over the time in treatments (weeks). The average bacteriological positivity (±SD) was 96.7 ± 4.6, 82.6 ± 9.2, 44.6 ± 23.1, 4.3 ± 0, and 6.5 ± 3.04 at baseline (week 0), weeks 2, 8, 22, and 26, respectively.

The overall patients with detectable TB symptoms at diagnosis were 97.8, 80.4, 86.9, 76, and 100% for cough, chest pain, sweats, loss of appetite, and sputum production, respectively, and matched with bacteriological positivity of 84.8, 84.7, 93.5, and 100% by SM, LJ culture, MGIT culture, and TB-MBLA, respectively. The proportion of patients with three clinical symptoms, such as night sweats, loss of appetite, and chest pain declined sharply to 9.1, 6.8, and 30.8%, respectively, by week 8 of treatment compared to bacteriological test positivity of 8.6, 17.4, 28.3, and 60.9% by sputum SM, LJ culture, MGIT culture, and TB-MBLA, respectively. No patients reported sweats at week 26 of treatment and only 13.3 and 4.4% had chest pain and loss of appetite, respectively. In contrast, cough resolution was slower than both MGIT culture and TB-MBLA (Figure 2) or average bacteriological positivity throughout treatment (Figure 3), except at week 8 with 50% positivity cough compared to 60.9% of TB-MBLA. Similar to cough, sputum production resolved more slowly than all bacteriological tests except TB-MBLA clearance at weeks 2 and 8 with a positivity of 89.1 and 60.9% compared to 82.6 and 56.8% for sputum production, respectively. A total of seven (15.2%) and 10 patients (21.7%) were coughing and producing sputum at week 26 of treatment, respectively, compared to four (8.6%) patients with TB-MBLA and two (4.3%) patients with MGIT positive. Only two out of the seven coughing patients at week 26 were among the six patients who were treatment failures.

### Agreements between clinical symptoms and bacteriological measures

At the time of diagnosis, there was more than 70% agreement between TB-MBLA, other bacteriological measures, and clinical symptoms. Cough, loss of appetite, and sputum production were 100% in agreement with TB-MBLA. Apart from cough and sputum production that was over 70% in agreement with TB-MBLA by week 2 of treatment, chest pain, sweats, and loss of appetite had significantly resolved, which increased discordance with TB-MBLA by over 60% (Table 2). Resolution of cough was consistent with bacteriological positivity score with a SLOPE of −3.2 and −3.5%, respectively, per week. The lowest agreement of TB-MBLA with clinical symptoms and other bacteriological tests was at week 8 between 32–44 and 45–59%, respectively, and increased to 71–87 and 82–89%, respectively, by the end of treatment week 26 (Table 2). Bacteriological positivity was not associated with the presence of TB symptoms at week 8 of treatment, *p* > 0.05 (Supplementary Table 1).

**TABLE 2:** Percentage (%) agreement of clinical symptoms with bacteriological tests.

	Agreement (%) throughout treatment					
	Variable	Baseline	Week 2	Week 8	Week 22	Week 26
Smear	Cough	86.96	41.30	53.49	68.29	86.67
	Chest pain	80.43	50.00	69.77	90.00	86.67
	Sweats	76.09	56.52	90.70	95.00	97.78
	Loss of appetite	73.91	63.04	86.05	87.50	97.78
	Sputum production	86.96	52.17	40.00	60.00	77.78
LJ culture	Cough	86.96	43.48	41.86	68.29	82.22
	Chest pain	80.43	43.48	53.49	90.00	82.22
	Sweats	80.43	58.70	72.09	95.00	93.33
	Loss of appetite	80.43	54.35	67.44	87.50	93.33
	Sputum production	86.96	36.96	33.33	60.00	73.33
MGIT culture	Cough	93.48	86.96	73.26	70.73	88.89
	Chest pain	78.26	34.78	55.81	87.50	84.44
	Sweats	82.61	34.78	72.09	92.50	91.11
	Loss of appetite	76.09	28.26	62.79	85.00	91.11
	Sputum production	93.48	67.39	62.79	62.50	75.56
TB-MBLA	Cough	100	82.61	44.19	65.85	75.56
	Chest pain	84.78	39.13	32.56	82.50	80.00
	Sweats	89.13	32.61	39.53	87.50	86.67
	Loss of appetite	100	30.43	34.88	80.00	86.67
	Sputum production	100	71.74	37.78	62.50	71.11
	Smear	86.96	54.35	45.65	88.37	88.89
	LJ culture	86.96	56.52	54.35	88.37	86.67
	MGIT culture	93.48	82.61	58.70	90.70	82.22

The percentage agreement between TB-MBLA with other bacteriological measures and clinical symptoms was over 80% at the time of diagnosis. Cough, loss of appetite, and sputum production were 100% in agreement with TB-MBLA. The percentage agreement decreased between treatment weeks 2 and 8, then increased between treatment weeks 22 and 26. The lowest agreement of TB-MBLA with all clinical symptoms and other bacteriological measures was at week 8 of treatment.

### Effect of bacillary load on tuberculosis symptoms and bacteriological test positivity

The median BL at baseline was 7.46E+05 eCFU/ml. A total of 23 patients (50%) had high BL above the median value (Table 3). Chest pain, night sweats, and loss of appetite resolved rapidly in response to treatment irrespective of baseline BL, *p* > 0.05. Cough resolved slowly and independently of the pre-treatment BL, *p* = 0.95. In contrast to cough, sputum production resolved more slowly in patients with low BL than those with high BL, *p* = 0.04. In a multivariate analysis, there was no association between low or high BL with the resolution of cough [AHR, 0.85 (0.42–1.73); *p* = 0.65], sputum production [AHR, 1.77 (0.90–3.47); *p* = 0.09], and chest pain [AHR, 1.25 (0.60–2.59); *p* = 0.55] after adjustment for HIV, age, and sex (Supplementary Table 2).

**TABLE 3:** Time to the resolution of clinical and bacteriological measures stratified by bacterial loads.

	Median time to resolution of clinical symptoms (weeks)			
Clinical symptoms	All patients (*n* = 46)	High-bacterial load (*n* = 23)	Low-bacterial load (*n* = 23)	*P*-value
Cough, *n* (IQR)	17.9 (8.0–23.9)	17.9 (8.0–20.5)	19.8 (8.0–23.9)	0.95
Sputum production, *n* (IQR)	19.4 (8.0–21.8)	8.1 (2.0–19.9)	19.9 (8.0–23.9)	0.04
Chest pain, *n* (IQR)	2.1 (2.0–19.8)	2.0 (2.0–11.5)	2.4 (2.0–19.9)	0.39
Sweats, *n* (IQR)	2.0 (2.0–8.0)	2.0 (2.0–8.0)	2.0 (2.0–6.0)	0.33
Loss of appetite, *n* (IQR)	2.0 (2.0–6.0)	2.0 (2.0–8.0)	2.3(2.0–6.0)	0.58
Smear microscopy, *n* (IQR)	7.8 (2.0–8.0)	2.1 (2.0–8.0)	8.0(2.0–8.0)	0.67
LJ, *n* (IQR)	7.8 (2.0–8.1)	8.0 (2.8–18.4)	2.0 (2.0–8.0)	0.03
MGIT, *n* (IQR)	8.0 (7.8–18.0)	8.0 (7.8–18.4)	8.0 (7.8–8.0)	0.83
TB-MBLA, *n* (IQR)	19.8 (8.0–19.9)	19.9 (11.7–19.9)	8.1 (7.8–19.9)	0.39

Cox proportion hazard ratio for the association between median time to the resolution (in weeks) of TB clinical symptoms stratified by the baseline bacterial load. IQR, interquartile range; LJ, Lowenstein–Jensen media; MGIT, mycobacterium growth indicator tubes; TB-MBLA; tuberculosis-molecular bacterial load assay.

**FIGURE 4:**
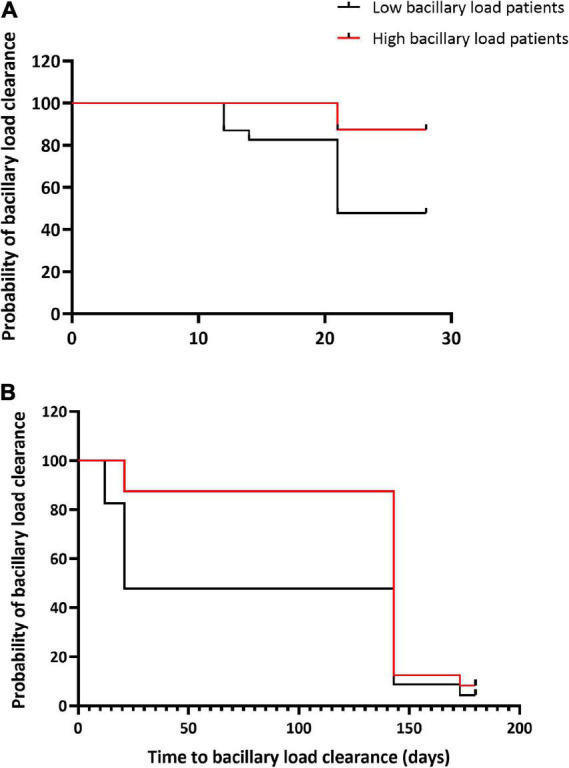
Bacillary load clearance during treatment in relation to baseline bacterial loads. Kaplan–Meier curves show TB-MBLA bacillary load clearance by week 8 of treatment **(A)** and by the end of treatment week 26 **(B)** among patients with high and low baseline bacterial load. Low baseline bacterial load patients were more likely to clear bacillary load at week 8, HR 5.6, *p* = 0.003 and at week 26 of treatment, HR 3.2, *p* = 0.014 than high baseline bacterial load patients.

The median time to sputum smear and LJ culture conversion-to-negative was shorter than the time for MGIT culture negative, while TB-MBLA had the highest median time to a negative result for patients with both low and high BL (Table 3). Unlike SM and MGIT culture, patients with low BL had a significantly shorter median time for conversion-to-negative, 2 weeks versus 8 weeks in patients with high BL, *p* = 0.03. Patients with low BL were more likely to clear TB bacillary load at weeks 8 and 26 of treatment, HR 5.6, *p* = 0.003 and HR 3.2, *p* = 0.014, respectively (Figure 4).

### Effect of HIV status on tuberculosis symptoms and bacteriological test positivity

The prevalence of HIV was 39.1%. Chest pain, night sweats, and loss of appetite resolved rapidly in response to treatment and there was no difference in the median time to the resolution of TB symptoms between patients with HIV positive and negative, *p* > 0.05 (Table 4). Overall, the median time to the resolution of cough and sputum production was slightly higher in patients with HIV positive than HIV negative, *p* > 0.05. For bacteriological tests, with the exception to SM, the median time to MGIT and LJ culture conversion from positive to negative was not statistically different among patients with HIV positive and negative, *p* > 0.05. Overall, TB-MBLA had the highest time to BL clearance among patients with HIV positive and negative compared to smear and culture tests, *p* > 0.05 (Table 4).

**TABLE 4:** Time to the resolution of TB symptoms and bacteriological tests stratified by HIV status.

	Median time to resolution of clinical symptoms (weeks)			
Clinical symptoms	All patients (*n* = 46)	HIV positive (*n* = 23)	HIV negative (*n* = 23)	*P*-value
Cough, *n* (IQR)	17.9 (8.0–23.9)	19.9 (9.8–23.9)	8.0 (7.8–23.9)	0.33
Sputum production, *n* (IQR)	19.9 (8.0–23.9)	19.9 (8.1–23.9)	8.0 (8.0–21.8)	0.67
Chest pain, *n* (IQR)	2.0 (2.0–11.5)	8.0 (2.0–19.9)	2.0 (2.0–8.0)	0.09
Sweats, *n* (IQR)	2.0 (2.0–8.0)	2.0 (2.0–11.5)	2.0 (2.0–2.8)	0.07
Loss of appetite, *n* (IQR)	2.0 (2.0–8.0)	2.0 (2.0–6.0)	2.0(2.0–8.0)	0.52
Smear microscopy, *n* (IQR)	8.0 (2.0–8.0)	2.1 (2.0–8.0)	8.0(2.0–8.0)	0.60
LJ, *n* (IQR)	6.0 (2.0–8.1)	2.0 (2.0–17.9)	8.0 (2.0–8.0)	0.60
MGIT, *n* (IQR)	8.0 (7.8–18.0)	8.0 (7.8–17.9)	8.0 (7.8–8.0)	0.84
MBLA, *n* (IQR)	19.8 (8.0–19.9)	18.4 (8.0–19.9)	19.8 (8.0–19.9)	0.86

Cox proportion hazard ratio shows the association between median time to resolution (in weeks) of TB clinical symptoms stratified by HIV status. *n* (IQR), number of weeks (interquartile range); LJ, Lowenstein–Jensen media; MGIT, mycobacterium growth indicator tubes; TB-MBLA; tuberculosis-molecular bacterial load assay.

## Discussion

Using the data from this clinical study, we have demonstrated that the concordance of symptoms and bacteriological tests is high before and low after treatment initiation. The strong pre-treatment agreement is in line with a recent report from South Africa (11). While other symptoms improved rapidly in the first 2 weeks of treatment, cough and sputum production improved slowly than bacteriological tests, as previously reported (13, 14). The rapid resolution of symptoms such as chest pain, night sweats, and loss of appetite seems to reflect the early bactericidal activity of anti-TB therapy. Studies have shown that most actively growing bacilli are killed within the first 2 weeks of treatment leaving the dormant (hard to clear) bacilli to persist longer on treatment course (23, 37).

Cough resolution followed closely that of bacillary load measured by TB-MBLA. The cough-TB-MBLA resolution relationship observed was more pronounced than the relationship with standard culture and sputum SM. Early clinical trials have demonstrated an association of TB symptoms with bacteriological tests during treatment (14, 38). Most importantly, analysis of the data from four clinical trials showed that TB symptoms (fever and sweats) resolved rapidly in the first 8 weeks of treatment whereas cough resolved slowly and was present in 20% of patients at the end of treatment (14). This finding is consistent with our study in which 20 and 15% of patients were coughing at weeks 22 and 26 of treatment, respectively, of whom four out of seven patients (57%) were HIV positive. A total of 14.2% of the coughing patients at week 26 were TB-MBLA positive. The presence of residual inflammation or other ailments arising as TB bacteria are cleared may explain the high positivity of cough at the end of treatment.

Interestingly, patients with low baseline BL took longer time to resolve sputum production than those with high BL. Further analysis of these patients revealed that 44% were HIV positive, an indication that the persistent sputum production could be driven by other opportunistic ailments and not underlying TB bacillary load. It is long established that HIV infection increases the risk of infection with other respiratory pathogens that may exacerbate and prolong symptoms such as cough and sputum production (39, 40). Thus, this interpretation and the overall relationship of clinical symptom clearance in TB/HIV-coinfected patients including those with low bacillary load warrants further investigation in a large cohort of HIV/TB-coinfected patients.

We further compared our results with a clinical trial for shortening the treatment of patients with drug-sensitive HIV-negative PTB conducted in Uganda, Brazil, and the Philippines. The study revealed that clinical symptoms declined rapidly by week 8 of treatment and that symptoms including fever, cough, and chest pain were more common among patients who had culture positive during follow-up (13). In contrast, the presence of TB symptoms at week 8 of treatment was not associated with bacteriological positivity in our study. Most patients with TB symptoms such as cough and sputum production at week 8 were negative by culture and sputum smear tests. Moreover, bacteriological tests were positive in some patients who had no TB symptoms at week 8 of treatment. At week 26, two patients who had no TB symptoms would have been declared clinically well and discharged from treatment in the absence of culture as the case in resource-poor countries. The novel TB-MBLA demonstrated highest positivity rate among patients with and without TB symptoms including those who were culture and smear negative. This discrepancy may be explained by the low sensitivity of SM, which is limited to 10,000 CFU/ml (6, 17), the effect of chemical decontamination on viable TB bacilli, and the presence of non-culturable bacteria, which do not grow in routine culture media (17, 21).

It is important to note that both clinical and bacteriological measures responded to treatment, falling to less than 20% positivity except for sputum production which was slightly above 20% positivity. The discordance could be due to the limitation of sputum-based tests as they only capture a proportion of bacilli in the lungs and only those that can growth in culture (41). This may explain why TB-MBLA which measures total RNA reflecting both actively growing and dormant bacilli is more sensitive and appears more consistent with the resolution of cough. Moreover, clinical symptoms are not specific and can be caused by any other respiratory disease. For example, cough and chest pain are some of the most common symptoms of COVID-19 (42).

Our study has several limitations. Screening and enrollment were based on the presence of TB symptoms at diagnosis and a positive Xpert MTB/RIF Assay result. This approach selectively included only patients who had symptoms of active PTB and increased the chance of those patients being positive by bacteriological tests at diagnosis and a higher percentage agreement than in treatment follow-up visits. Secondly, we enrolled a low number of patients with 6-month follow-up visits and we were not able to establish if treatment failure was due to relapse or re-infections. Because of the small number of patients, we were unable to find a difference in time to the resolution of TB symptoms or bacteriological positivity in relation to baseline BL and HIV status. Clinical studies involving larger number of patients and longer follow-up past the current 6 months of TB therapy in healthcare settings are underway and will shed more light on the long-term relationship of clinical symptoms particularly cough with bacteriological tests and final outcomes. Such studies will further help to understand the clinical relevance of TB positive results by TB-MBLA when routine standard bacteriological tests, culture, and microscopy are negative and provide additional data about the causes of recurrent tuberculosis after successful treatment.

## Conclusion

We showed a high percentage agreement of clinical symptoms with bacteriological positivity for TB at diagnosis which weakens rapidly during the early weeks of anti-TB therapy. Our findings provide new evidence that relying solely on TB symptoms for diagnosis or monitoring may mislead treatment decisions of some patients and final treatment outcomes. Our findings advocate for more investments in bacteriological tests to improve the accuracy of TB diagnosis and treatment monitoring in routine healthcare settings.

## Data availability statement

The data supporting the conclusions of this article will be available by authors upon request and without undue reservation.

## Ethics statement

The studies involving human participants were reviewed and approved by the Mbeya Medical Research and Ethics Committee (MRH/R.10/18VOLL.VII/12) and University of St Andrews Teaching and Research Ethics Committee (MD12678). National approval was obtained from National Institute for Medical Research (NIMR/HQ/R.8a/Vol.IX/2400) in Tanzania. The patients/participants provided their written informed consent to participate in this study.

## Author contributions

BM, WS, NN, IS, and SG conceived and designed the study. BM, WS, IS, and NN developed the research tools. BM, ES, and JJ collected the data. BM, WS, and WO led database cleaning, data analysis, and created figures and tables. BM drafted the manuscript. SG, WS, and NN proofread the manuscript. All authors reviewed the manuscript before submission.
