# Supplementary material for: Systematic assessment of clinical and bacteriological markers for tuberculosis reveals discordance and inaccuracy of symptom-based diagnosis for treatment response monitoring
Source: Front Med (Lausanne). 2022 Oct 28;9:992451. doi: 10.3389/fmed.2022.992451 (PMC9677322; doi:10.3389/fmed.2022.992451)
Supplement: Supplementary file 1 [file Data_Sheet_1.docx]

**Supplementary materials:**

***Supplementary table 1:*** Association of TB symptoms at week 8 with bacteriological tests

|  |  | Microscopy | | | MGIT culture | | | LJ culture | | | TB-MBLA | | | |
| --- | --- | --- | --- | --- | --- | --- | --- | --- | --- | --- | --- | --- | --- | --- |
|  |  | Positive | Negative | *P* | Positive | Negative | *P* | Positive | Negative | *P* | Positive | Negative | *P* |  |
| Cough | Present, n (%) | 3(13) | 20(87) | 0.23 | 6(26.1) | 17(73.9) | 1.00 | 4(17.4) | 19(82.6) | 1.00 | 13(56.5) | 10(43.5) | 0.53 |  |
|  | Absent, n (%) | 0(0) | 20(100) |  | 6(30) | 14(70) |  | 4(20.0) | 16(80.0) |  | 13(65) | 7(35) |  |  |
| Sputum production | Present, n (%) | 2(7.4) | 25(92.6) | 1.00 | 5(18.5) | 22(81.5) | 0.09 | 3(11.1) | 24(88.9) | 0.23 | 14(51.9) | 13(48.1) | 0.05 |  |
|  | Absent, n (%) | 2(11.1) | 16(88.9) |  | 8(44.4) | 10(55.6) |  | 5(29.4) | 12(70.6) |  | 14(77.8) | 4(22.2) |  |  |
| Chest pain | Present, n (%) | 2(14.3) | 12(85.7) | 0.24 | 4(28.5) | 10(71.4) | 1.00 | 2(14.3) | 12(85.7) | 1.00 | 6(42.9) | 8(57.1) | 0.09 |  |
|  | Absent, n (%) | 1(3.4) | 28(96.6) |  | 8(27.6) | 21(72.4) |  | 6(20.7) | 23(79.3) |  | 20(68.9) | 9(31.0) |  |  |

Presence of TB symptoms at week 8 was not associated with bacteriological test positivity; Fischer exact test, p>0.05. Cough at week 8 (present, n=23; absent, n=20); Sputum production (present, n=27; absent, n=18); Chest pain (present, n=14; absent, n=29)**. Abbreviations:** LJ, Lowenstein Jensen media; MGIT, Mycobacterial Growth Indicator Tubes; TB-MBLA, Tuberculosis Molecular Bacterial Load Assay. **Definitions:** MGIT/LJ positive, Culture media that had positive growth for *M. tuberculosis* confirmed by MPT64 antigen test; MGIT/LJ negative, Culture media that had no growth for *M. tuberculosis* after incubation for 42 days for MGIT and 56 days for LJ culture at 37°C including culture that *M. tuberculosis* could not be confirmed by MPT64 antigen test.

***Supplementary table 2:***Association between baseline TB-MBLA bacterial load and time to resolution of clinical symptoms.

|  | **Cough** | | **Sputum production** | | **Chest Pain** | |
| --- | --- | --- | --- | --- | --- | --- |
|  | AHR [95% CI] | P-value | AHR [95% CI] | P-value | AHR [95% CI] | P-value |
| **Baseline bacterial load**  Low  High | Ref  0.85(0.42-1.73) | 0.65 | Ref  1.77 (0.90-3.47) | 0.09 | Ref  1.25 (0.60-2.59) | 0.55 |
| **HIV**  Negative  Positive | Ref  0.71 (0.35-1.46) | 0.36 | Ref  1.08 (0.51-2.26) | 0.85 | Ref  0.59 (0.27-1.31) | 0.19 |
| **Sex**  Male  Female | Ref  0.52 (0.23-1.17) | 0.12 | Ref  0.85 (0.42-1.72) | 0.65 | Ref  1.27 (0.59-2.73) | 0.54 |
| Age | 1.01(0.97-1.03) | 0.84 | 0.99(0.97-1.02) | 0.66 | 0.98 (0.95-1.01) | 0.26 |

Multivariate Hazard Ratio and 95% confidence interval estimating association between baseline TB-MBLA bacterial load and time to resolution of clinical symptoms. No association found in baseline bacterial load and resolution of sputum production, chest pain and cough after adjusting for HIV status, age, and sex.

***Supplementary table 3:*** Relationship of baseline AFB smear grade and Xpert MTB/RIF with clinical symptoms

|  | TB symptoms | | | | | |
| --- | --- | --- | --- | --- | --- | --- |
|  |  | Cough, n (%) | Sweats, n (%) | Chest pain, n (%) | Loss of appetite, n (%) | Sputum production, n (%) |
|  | Negative | 7(15.2) | 7(17) | 5(12.8) | 4(11.1) | 7(15.2) |
| Smear microscopy | Scanty | 2(4.4) | 2(4.9) | 2(5.1) | 1(2.8) | 2(4.4) |
|  | 1+ | 5(10.9) | 5(12.2) | 5(12.8) | 4(11.1) | 5(10.9) |
|  | 2+ | 6(13) | 5(12.2) | 5(12.8) | 5(13.9) | 6(13) |
|  | 3+ | 26(56.5) | 22(53.7) | 22(56.4) | 22(61.1) | 26(56.5) |
|  |  |  |  |  |  |  |
| Xpert MTB/RIF Assay | High | 25(54.3) | 21(51.2) | 22(56.4) | 20(55.6) | 25(54.3) |
|  | Medium | 13(28.3) | 12(29.3) | 11(28.2) | 11(30.6) | 13(28.3) |
|  | Low | 5(10.9) | 5(12.2) | 3(7.7) | 4(11.1) | 5(10.9) |
|  | Very low | 3(6.5) | 3(7.3) | 3(7.7) | 1(2.7) | 3(6.5) |

A high proportion of patients with clinical symptoms were AFB positive by ZN smear microscopy and Xpert MTB/RIF Assay at baseline. For each clinical symptom studied, majority of patients had a high smear and Xpert MTB/RIF grades followed by the corresponding lower smear and Xpert semiquantitative grades. All patients were Xpert MTB/RIF Assay positive and a small proportion of patients with clinical symptoms were AFB smear negative.
